# Challenges in mimicking hypoxia: insights into HIF-regulated MiRNA expression induced by DMOG and CoCl_2_

**DOI:** 10.1186/s12964-025-02459-7

**Published:** 2025-10-22

**Authors:** Anna Barton, Maciej Jaśkiewicz, Anna Więch -Walów, Adrianna Moszyńska, Aleksandra Cabaj, Ben Wielockx, Liliana Schaefer, James F. Collawn, Rafal Bartoszewski

**Affiliations:** 1 Department of Biophysics, Faculty of Biotechnology, University of Wroclaw, F. Joliot-Curie 14a, Wroclaw, 50- 383 Poland; 2 Laboratory of Photobiology and Molecular Diagnostics, Intercollegiate Faculty of Biotechnology, University of Gdansk and Medical University of Gdansk, Gdańsk, Poland; 3 Laboratory of Sequencing, Nencki Institute of Experimental Biology of the Polish Academy of Sciences, Warsaw, Poland; 4 Institute of Clinical Chemistry and Laboratory Medicine, University Carl Gustav Carus and Medical Faculty, Technische Universität Dresden, Fetscherstrasse 74, Dresden, 01307 Germany; 5 Institute of Pharmacology and Toxicology, Goethe University Frankfurt, Frankfurt, Germany; 6 Department of Cell, Developmental and Integrative Biology, University of Alabama at Birmingham, Birmingham, AL 35233 USA

**Keywords:** *HIF1A*, *EPAS1*, HIF-1α, HIF-2α, Hypoxia mimetics, MicroRNA

## Abstract

The cellular and tissue response to acute hypoxia is mediated by a complex adaptive response initiated by hypoxia-inducible factors (HIF) 1 and 2. HIF proteins are key transcription factors that modulate gene expression to adapt cells to the low oxygen tension. Cellular studies of hypoxia have used the low oxygen model by using hypoxia chambers to monitor the gene expression changes, while others have utilized hypoxia mimetics in normoxic conditions. Here, we provide a comparison of the utility and limitations of two hypoxia mimetics by studying the changes in the miRNA and mRNA expression profiles compared to the low oxygen model. We utilized data from NGS-based global analyses, qPCR validation, and HIFa silencing experiments to determine the distinct profiles of HIF-1 and HIF-2 activities of the hypoxia and mimetic models. The ability of CoCl_2_ and DMOG (dimethyloxalylglycine) to mimic hypoxia was tested in cultured human endothelial cells. The results demonstrated that their treatments resulted in a differential stabilization of HIF-1α and HIF-2α, with CoCl_2_ predominantly stabilizing HIF-1α, and DMOG stabilizing HIF-2α. While CoCl_2_ and DMOG show potential in mimicking certain isoform-specific effects, their inability to completely mimic their effects on miRNAs and their mRNA targets reveals that these changes do not fully reproduce the hypoxia-induced effects in pathways related to angiogenesis and apoptosis. Taken together, while the hypoxia mimetics offer valuable insights into the isoform-specific effects of HIF-1 and HIF-2, their inability to fully replicate the complexity of hypoxia-induced miRNA interactions is not illustrated in human endothelial cells.

**Supplementary Information:**

The online version contains supplementary material available at 10.1186/s12964-025-02459-7.

## Introduction

The ability to detect and adapt to fluctuations in oxygen levels is vital for the survival of aerobic organisms [1]. Induction of hypoxia activates a complex adaptive response mediated by hypoxia-inducible factors 1 and 2 (HIF-1 and − 2), which act as central regulators that link low oxygen conditions to changes in gene expression [2–5]. While these transcription factors play important roles in critical physiological processes such as embryogenesis and cell differentiation, alterations in the adaptive responses have been implicated in diseases like coronary artery disease, stroke, cancer, and chronic obstructive pulmonary disease [6–9].

Another function of the hypoxic adaptive response is in its regulation by miRNAs that modulate the transition between HIF-1 and HIF-2 signaling during prolonged hypoxia [10]. Research is urgently needed to understand these pathways in humans to advance medicine. Most research is limited to hypoxia models in cell culture, which have been instrumental in uncovering the cellular, biochemical, and molecular mechanisms of the hypoxia response. Among these methods, the low oxygen-induced hypoxia model remains the gold standard due to its ability to closely mimic physiological conditions. However, the accessibility of hypoxia chambers or CO_2_ incubators with controlled oxygen levels presents a significant challenge for many laboratories. The intermittent reintroduction of oxygen when using CO_2_ incubators further complicates experimentation. While larger hypoxia processing chambers with glove boxes provide a continuous hypoxic environment conducive to media changes and cell manipulation, their prohibitive cost limits their availability.

Consequently, the chemically induced hypoxia models have served as a practical alternative. These mimics have emerged as popular tools to replicate hypoxic conditions by stabilizing HIF-α subunits, offering an accessible, yet imperfect solution for investigating the hypoxia response. The CoCl_2_-induced chemical hypoxia model is one of the most widely used hypoxia mimetics due to its ability to stabilize HIF-α under normoxic conditions [11]. Mechanistically, it is widely accepted that Co^2+^ replaces Fe^2+^ in prolyl hydroxylases (PHDs), enzymes that mediate HIF-subunits degradation under normoxic conditions. This substitution impairs PHDs activity, allowing HIF-α stabilization even in the presence of oxygen. However, other mechanistic explanations for CoCl_2_-related accumulation of HIF-α has been also proposed (as reviewed in [11]). Notably, although cobalt ions effectively inhibit prolyl hydroxylation and thereby suppressing all three PHD isoforms, they have limited capacity to inhibit asparaginyl hydroxylation, and therefore have a minimal impact on FIH activity [12]. Historically, CoCl₂ was used to treat anemia due to its ability to stimulate erythropoiesis [13, 14]. However, its use has been largely discontinued because of its toxicity and serious side effects [15].

The other chemical model, DMOG, acts as a competitive inhibitor of all three PHD isoforms and FIH, and serves as an analogue of 2-oxoglutarate, which is a co-substrate for PHDs. By occupying the catalytic site, DMOG blocks enzymatic activity, thereby preventing HIF-α degradation in normoxia [16]. However, the presence of oxygen during the treatments with the mimetics raises concerns about the extent to which results obtained using these models accurately reflect the true hypoxic responses. Furthermore, the hypoxia mimetics have been shown to display pleiotropic dose-, time-, and cell type-specific effects that include among others induction of cell death [11].

Although hypoxia mimetics have proven their usefulness in some of the early HIF-oriented research, many concerns were also raised regarding their ability to effectively mimic hypoxia in vitro and in vivo [11, 17–19]. In this study, we compared the effects of the two mimetic models, CoCl_2_ and DMOG models to the low oxygen model in terms of their impact on miRNA and mRNA regulatory networks. The results indicate that while the general changes in the miRNA global expression profiles were somewhat correlated with those obtained with hypoxia, and both CoCl_2_ and DMOG treatments partially resembled HIF-1-related reprogramming of miRNA profiles during hypoxia, neither of tested hypoxia mimetics were able to properly replicate the specific roles of HIF-1 and HIF-2 on the miRNA expression profiles. Furthermore, both DMOG and CoCl_2_ treatments underrepresented HIF-1- and HIF-2-mediated posttranscriptional impact on the cellular adaptation to hypoxia, including their modulation of angiogenic signaling.

Taken together, this study highlights the limitations of hypoxia mimetics as a model for testing both hypoxia and the HIF impact on miRNA expression as well as the posttranscriptional aspects of the adaptive response to hypoxia.

## Results

**Fig. 1:**
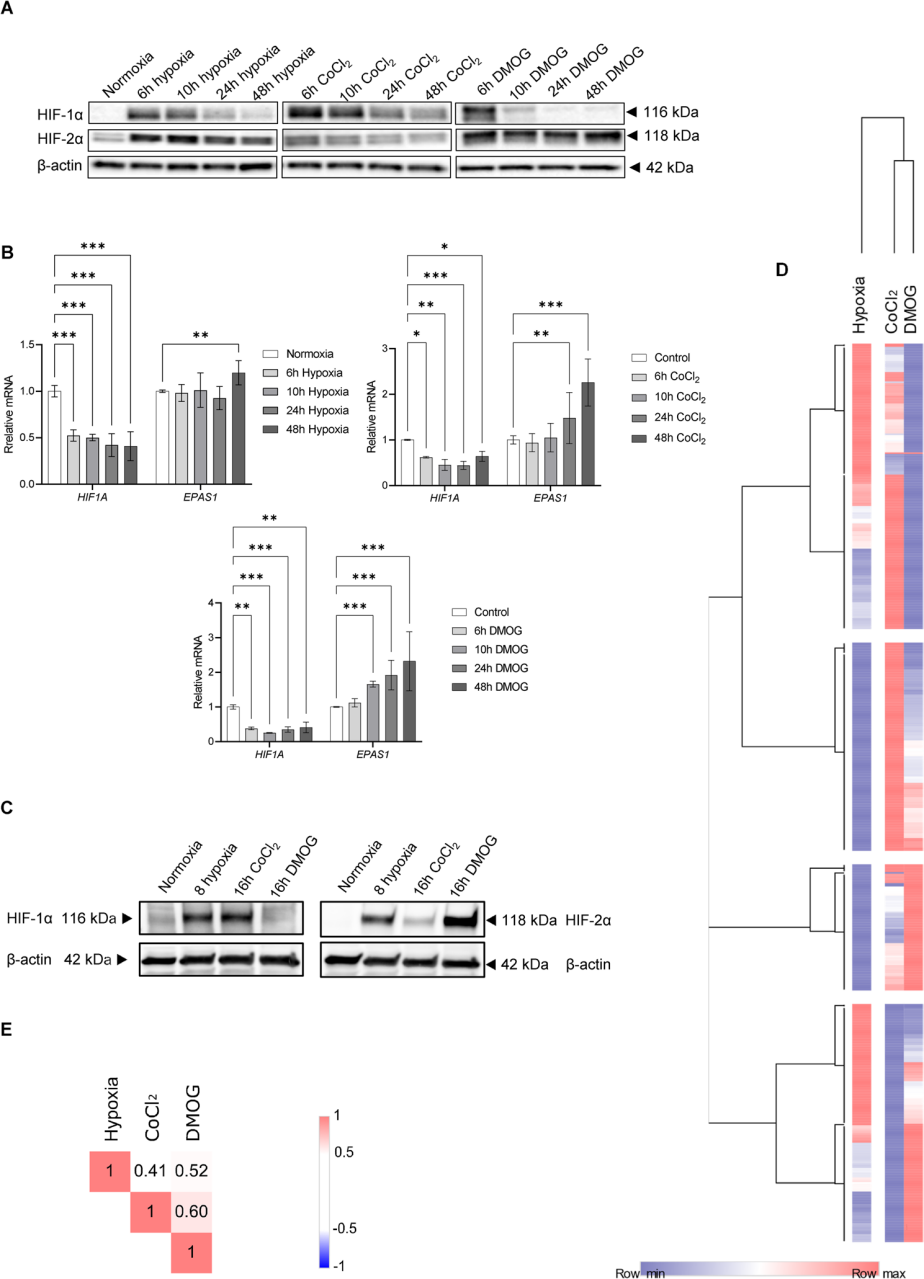
Hypoxia mimetics do not reflect the kinetics of accumulation of HIF-1α and HIF-2α in HUVECs. **A** The representative changes in HIF-1α and HIF-2α protein levels were evaluated after exposure to hypoxia or treatments 200 µM CoCl_2_ and 1mM DMOG for the time periods specified by Western Blot. Uncropped blots are presented in Supplemental Fig. 1. **B** The corresponding changes in *HIF1A* and *EPAS1* mRNA levels were quantified by quantitative real-time PCR and normalized to *RPLP0* rRNA levels and expressed as a fold change over control. Data represents the mean ± SEM of three biological replicates (*P < 0.05, **P < 0.01 and ***P < 0.001 were considered significant). **C** HIF-1α and HIF-2α protein levels were evaluated after 8 h exposure to hypoxia and treatments with 200 µM CoCl_2_ or 1mM DMOG for 16 h by Western Blot and normalized to β-actin and total protein levels. **D** The heat map represents the general distribution of unique miRNAs affected during 8 h of hypoxia exposure and 16 h of treatment with CoCl_2_ and DMOG (Supplemental Data Set 1). At each time point the miRNAs levels were expressed as fold change of hypoxia exposed or treated samples compared to the normoxic and untreated control samples. The heatmap generation and hierarchical clustering were performed with the Morpheus Webserver (https://software.broadinstitute.org/morpheus). **E** Hypoxia, CoCl_2_ and DMOG differ in their effect on the HUVECSs’ genome-wide mRNA profiles. The global distributions of miRNA changes at each condition were compared with the Spearman rank–order correlations

To test the effectiveness of CoCl_2_ and DMOG as tools to study their impact on the miRNA-dependent regulatory networks, we compared the HIF-1α and HIF-2α protein levels in human umbilical vein endothelial cells (HUVECs, a 10-donor pool) over a 48-hour time course of hypoxia exposure (0.9% O_2_) and in CoCl_2_ and DMOG treatments (Fig. 1 A). Consistent with our previous findings, HIF-1α rapidly accumulated in HUVECs exposed to hypoxia for 6 to 10 h, and was dramatically reduced by 24 and 48 h [20]. HIF-2α, on the other hand, reached its maximum at 10 h and remained elevated up to 48 h. In contrast, the HUVECs exposed to CoCl_2_ accumulated HIF-1α from the 6 h-time point and remained elevated up to 48 h. Furthermore, HIF-2α was only modestly stabilized by CoCl_2_ (Fig. 1 A). The DMOG treatment resulted in an opposite pattern compared to CoCl_2_. DMOG stabilized HIF-1α only up to 10 h, whereas the DMOG-treated cells accumulated HIF-2α up to 48 h (Fig. 1 A). Taken together, this data shows that neither mimetic reproduces the hypoxia-related HIF-1α and HIF-2α accumulation nor the related HIF switch transition. It also demonstrated that CoCl_2_ is more specific for HIF-1α stabilization, whereas DMOG is more specific for HIF-2α.

We also measured the changes in mRNA of *HIF1A* (encoding HIF-1α) and *EPAS1* (encoding HIF-2α). As shown in Fig. 1B, all hypoxia, CoCl_2_ and DMOG treatments reduced *HIF1A* mRNA levels. Interestingly, prolonged treatments with both mimetics increased *EPAS1* expression, which did not correlate with HIF-2α accumulation during CoCl_2_ treatment. Furthermore, in HUVECs exposed to hypoxia, *EPAS*1 transcript levels remain stable, with a modest increase observed at 48 h, as previously reported [20, 21].

Based on these experiments, we concluded that because of the preference of these hypoxia mimetics for either HIF-1α or HIF-2α, they do not replicate hypoxic conditions well. However, they can be still a useful tool to study specific HIF-α isoform effects.

As shown in Fig. 1C, HUVECs exposed to CoCl_2_ for 16 h accumulate mainly HIF-1α at levels comparable to those observed upon 8 h exposure to hypoxia. In DMOG-treated cells, however, HIF-1α level remains low. Conversely, HIF-2α, is modestly accumulated in CoCl_2_-treated cells, while in the DMOG-treated ones, the level of this protein is higher than in hypoxia-exposed HUVECs (Fig. 1 C). Thus, we speculated that overnight (16 h) treatment with CoCl_2_ and DMOG would modulate HIF-1 and HIF-2 signaling responses, respectively.

To test the HIF-specific impact on global miRNA expression profiles, we isolated total RNA from cells cultured in normoxia and from cells exposed to hypoxia for 8 h to evaluate the effects of simultaneous HIF-1α and HIF-2α stabilization on miRNA expression. We also tested the cells treated with CoCl_2_ for 16 h to assess the impact of selective HIF-1α stabilization and the cells treated with DMOG for 16 h to examine the effects of stabilization of both HIF-1α and predominantly HIF-2α on miRNA expression. We selected the 8-hour hypoxia time point for HIFs knockdown experiments to balance biological relevance and experimental feasibility, as both HIF-1α and HIF-2α show comparable accumulation and exert significant effects on miRNA expression at this stage, while shorter exposures yield minimal changes and longer ones introduce secondary signaling [20, 22]. For the mimetic treatments, a 16-hour exposure was chosen based on availability of previously obtained gene expression and miRNA expression data under hypoxia at this time point [20, 22].

The samples were then subjected to genome-wide next generation sequencing (NGS) followed by bioinformatics analyses. As shown in Fig. 1D, all tested conditions led to extensive changes in global miRNA expression profiles. Interestingly, despite the notable correlation between treatments, the hypoxia mimetics induced changes in the miRNA expression profiles were more correlated to each other than to the hypoxia treatment (Fig. 1DE). The correlations in miRNA expression profiles suggest that while both hypoxia mimetics may have the potential to resemble the HIF-mediated reprogramming of miRNA networks, the differences may result from their different HIF-α preferences.

**Fig. 2:**
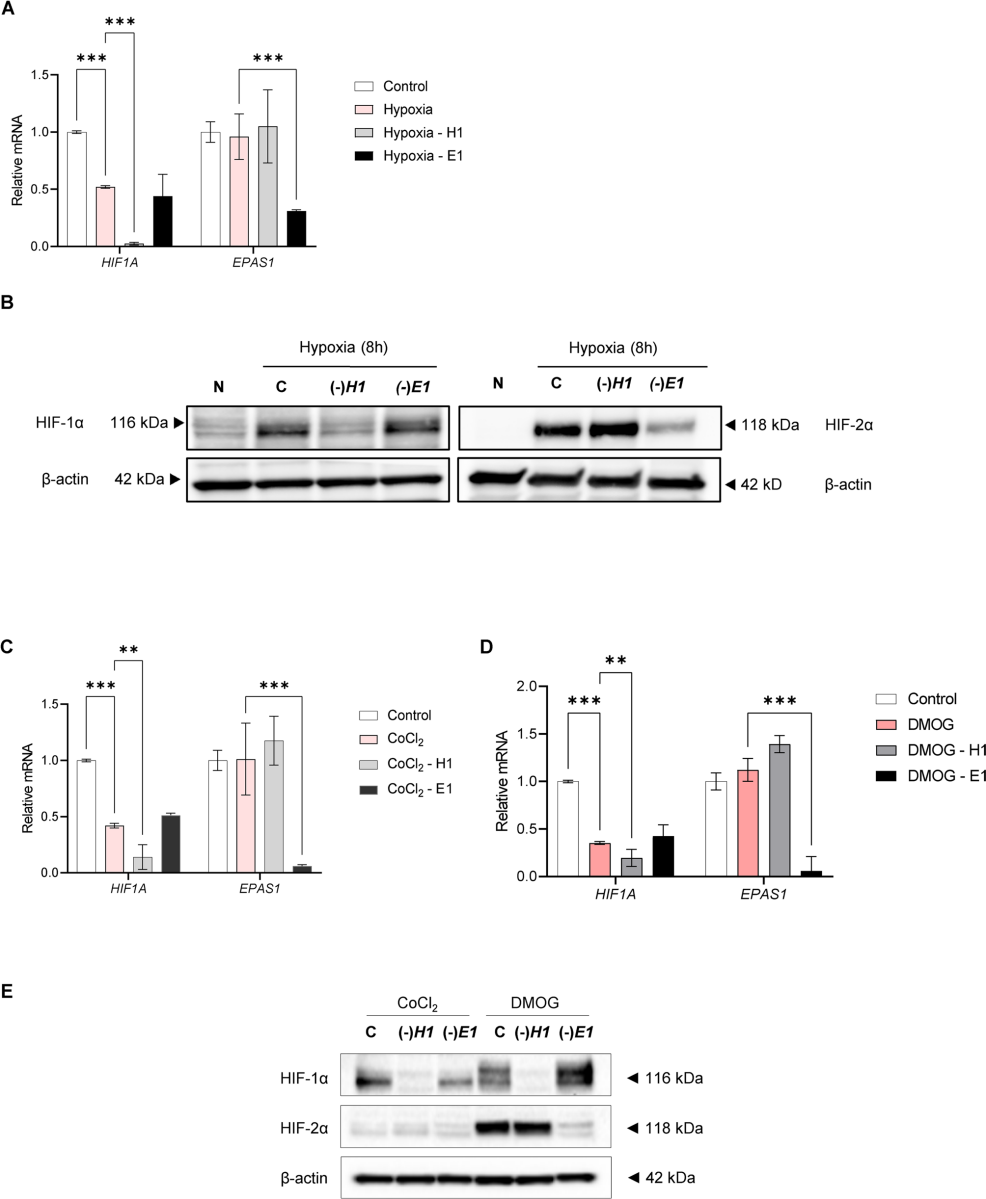
*HIF1A* and *EPAS1* silencing in HUVECs exposed to hypoxia. **A **The corresponding *HIF1A* and *EPAS1* mRNA levels were quantified by quantitative real-time PCR and normalized to *RPLP0* rRNA levels and expressed as a fold change over control at the specific time-point of hypoxia. Data represent the mean ± SEM of three biological replicates (*P < 0.05, **P < 0.01 and ***P < 0.001 were considered significant). **B** HIF-1α and HIF-2α protein levels were evaluated in normoxia and hypoxia, and by Western Blot and normalized to β-actin and total protein levels. N –normoxia; C – hypoxia, negative control siRNA; H1 – hypoxia, *HIF1A* siRNA; E1 – hypoxia, *EPAS1* siRNA. *HIF-1α and HIF-2α silencing in HUVECs exposed to CoCl*_*2*_ *and DMOG*. The corresponding *HIF1A* (**C**) and *EPAS1* (**D**) mRNA levels were quantified by quantitative real-time PCR and normalized to *RPLP0* rRNA levels and expressed as a fold change over control. Data represents the mean ± SEM of three biological replicates (*P < 0.05 was considered significant) (*P < 0.05, **P < 0.01 and ***P < 0.001 were considered significant). **E** HIF-1α and HIF-2α protein levels were evaluated after treatments 200 µM CoCl_2_ or 1mM DMOG for 16 h by Western Blot and normalized to β-actin and total protein levels and related to the control. N –normoxia, negative control siRNA; H1 – hypoxia, *HIF1A* siRNA; E1 – hypoxia, *EPAS1* siRNA, N*- DMOG/CoCl_2_ treated cells, negative control siRNA

To further test this hypothesis, we performed silencing experiments in HUVECs treated with both hypoxia mimetics, DMOG and CoCl_2_ and cells exposed to hypoxia. As shown in Fig. 2AB, *HIF1A* and *EPAS1* silencing in hypoxia-exposed HUVECs was efficient at both the protein and mRNA levels. Furthermore, although in some experiments we observed some increase in HIF-1α upon *EPAS1* knockdown, however, this phenomenon was not significant for the HIF-1α protein levels between three biological replicates [23]. The *HIF1A* silencing in CoCl_2_ treated HUVECs was also successful at both HIF-1α protein and mRNA levels, whereas the HIF-2α protein accumulation under these conditions was very low, and despite successful silencing of *EPAS1* mRNA, there was no significant impact on the HIF-2α protein levels (Fig. 2CE). This observation is in good agreement with previous reports showing that CoCl_2_ preferably leads to accumulation of HIF-1α rather than HIF-2α [24, 25]. DMOG treatments resulted in the same patterns of HIF-1α and HIF-2α as those observed in hypoxia-exposed cells (Fig. 2DE). Both HIF-α subunits accumulated upon DMOG treatment, and both efficiently silenced at the protein and mRNA levels. Furthermore, as observed in hypoxia, *EPAS1* silencing upregulated HIF-1α levels.

**Fig. 3:**
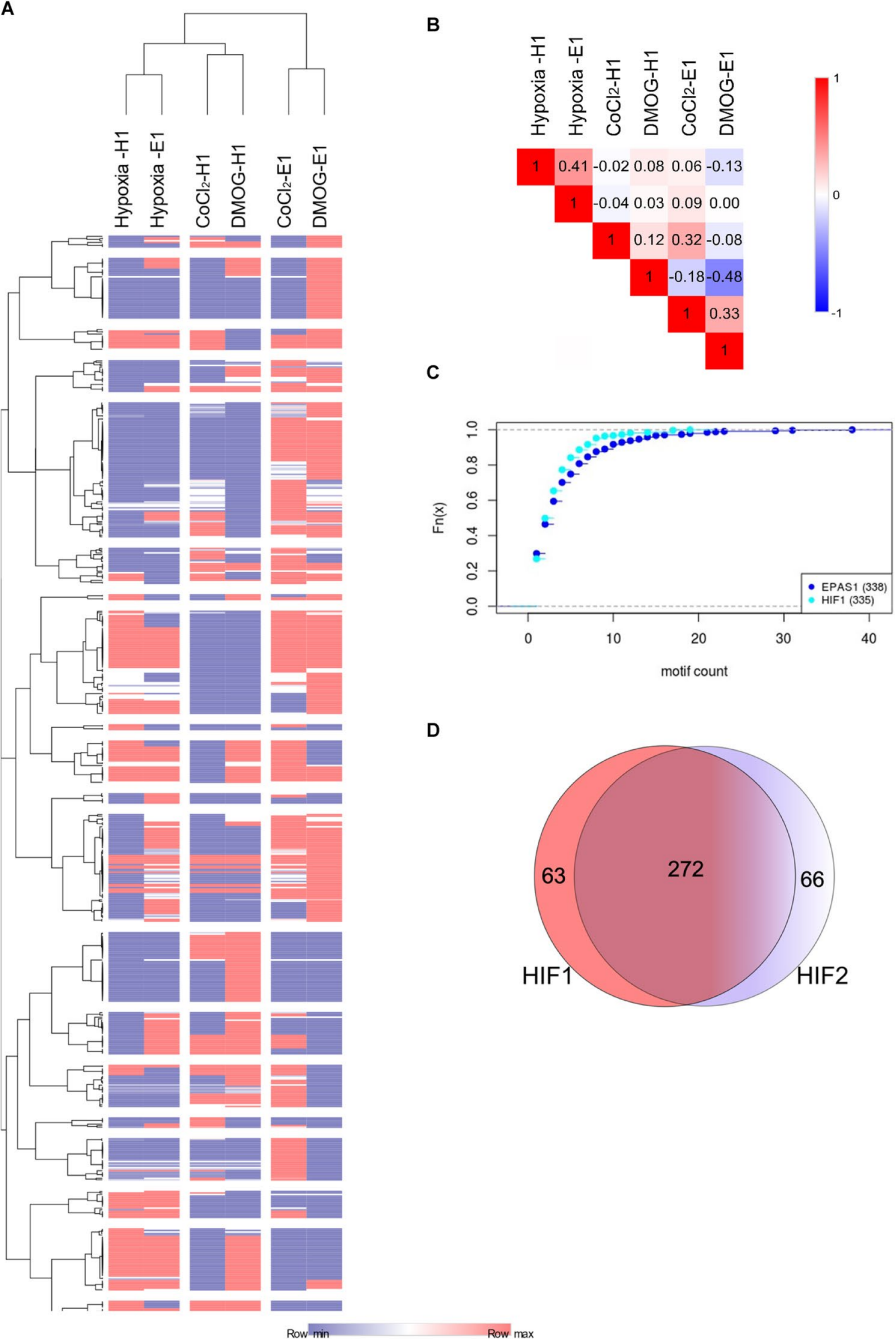
*HIF1A* and *EPAS1* silencing in HUVECs *exposed to hypoxia or hypoxia mimetics has different impact on global miRNA profiles*. **A** The heat map represents the general distribution of miRNAs affected by either *HIF1A* or *EPAS1* silencing during 8 h of hypoxia exposure and 16 h of treatment with CoCl_2_ and DMOG. The miRNA levels are represented as a fold change over control siRNA for each experimental condition (Supplemental Data Set 1). The heatmap generation and hierarchical clustering were performed with the Morpheus Webserver (https://software.broadinstitute.org/morpheus). **B** The global distributions of miRNA changes at each condition were compared with the Spearman rank–order correlations. **C** Cumulative distribution functions of counts of HRE binding motifs based on HOCOMOCO v.9 (HIF-1–specific and HIF-2–specific summed) per miRNA gene in open chromatin regions. **D** The Venn diagram represents the general distribution of HIF-1-specific and HIF-2-specific miRNAs (Supplemental Data Set 2)

Consequently, we performed next generation analysis in each of tested models (8 h hypoxia, CoCl_2_ treatment or DMOG treatment for 16 h) when the *HIF1A* or *EPAS1* was silenced. Although we were already aware that the mimetics were unlikely to fully replicate hypoxic impact of HIF-1α and HIF-2α on cellular miRNA profiles, based on the presented experimental workflow, we expected the results obtained from *HIF1A* silencing in CoCl_2_-treated cells and from *EPAS1* knockdown in DMOG-treated ones to correlate with those obtained for the respective knockdowns in hypoxia-exposed HUVECs. Notably, however, as shown in Fig. 3AB, despite some similarities, silencing of either *HIF1A* or *EPAS1* with both mimetic treatments resulted in specific miRNA expression patterns that were knockdown target-specific and this poorly corelated with their hypoxic counterparts. Previously, we demonstrated that in HUVECs exposed to hypoxia, although there were HIF-1- and HIF-2-specific miRNAs that were regulated, the majority of these noncoding RNAs were regulated by both [22]. This observation is further supported by this study, as we have observed a similar correlation between the miRNA profiles of *HIF1A* and *EPAS1* silencing. Nevertheless, the results of *HIF1A* and *EPAS1* knockdown were either poorly or negatively correlated with each other in the CoCl_2_ and DMOG treatments, respectively (Fig. 3AB).

Next, we examined the genomic locations of the miRNAs that were in open chromatin regions established in the HUVEC cell line by the ENCODE project for the presence of distinct hypoxia response element (HRE) motifs for HIF-1 and for HIF-2 (Fig. 3 C, Supplemental Data Set 2) [26–31]. As shown in Fig. 3D, this analysis confirmed that the vast majority of affected miRNAs can be regulated by both HIF-1 and HIF-2, whereas only a small number are HIF-α-isoform specific. Consequently, in hypoxia exposed cells, when both isoforms are present, silencing either one could be compensated by the other. In mimetics-treated cells, silencing the dominant isoform should have a more dramatic and HIF-α specific effect.

**Fig. 4:**
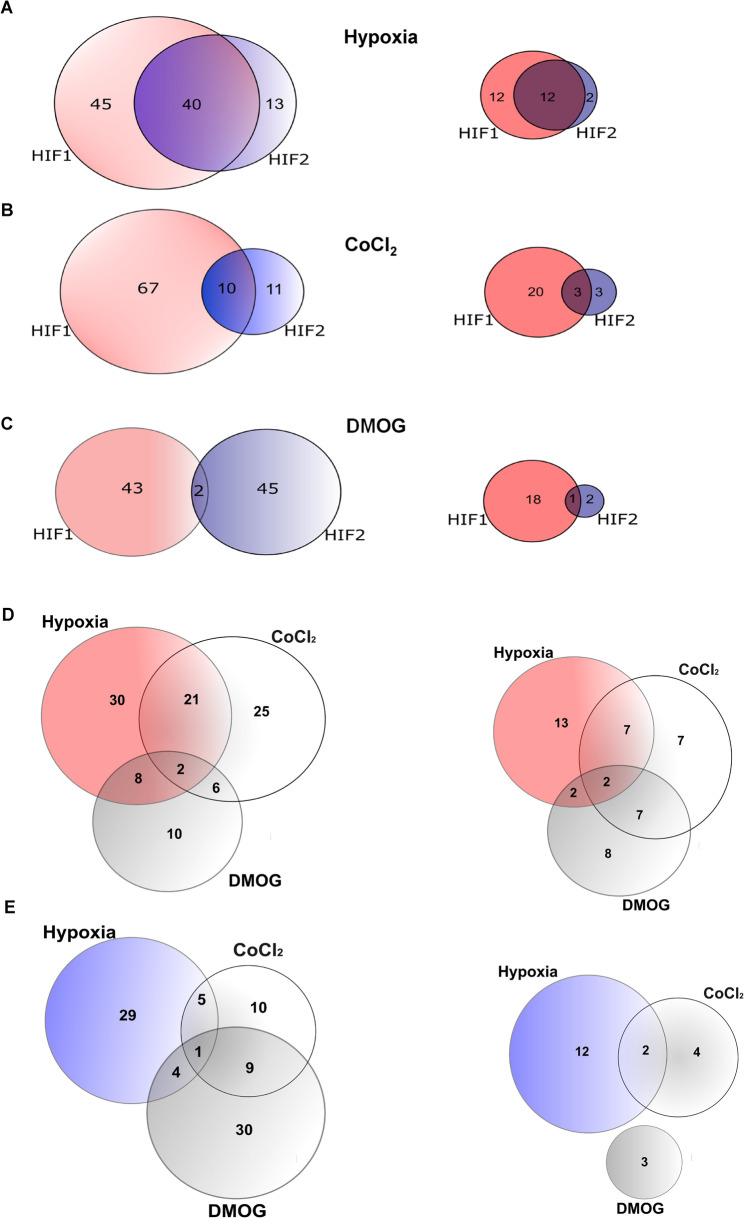
Hypoxia mimetics do not reflect HIF-1α and HIF-2α impact on miRNA expression in HUVECs. The Venn diagram on the left represents the NGS-based general distribution of all unique miRNAs levels that are reduced significantly upon *HIF1A* (red) and *EPAS1* (blue) silencing in HUVECs exposed and contained respective HRE motifs (right panel) to hypoxia for 8 h (**A**), CoCl_2_ for 16 h (**B**), and DMOG for 16 h (**C**). The comparison of hypoxia and hypoxia mimetics in relation to the reduction of specific miRNA levels associated with *HIF1A* (**D**) and *EPAS1* (**E**) is illustrated using Venn diagrams. The right panels highlight miRNAs that contain either HIF1A- or EPAS1-binding motifs

Interestingly, as shown in Fig. 4, the total number of miRNAs downregulated following HIF-1 or HIF-2 silencing is relatively consistent across CoCl₂ (*n* = 88), DMOG (*n* = 90), and hypoxia (*n* = 98). However, the gene expression profiles linked to HIF-1 and HIF-2 specific motifs vary significantly between these conditions. When we compared individual miRNA changes in hypoxia-exposed cells with HIF knockdowns, we noted that out of 85 miRNAs that were reduced when *HIF1A* was silenced, 40 were also downregulated when *EPAS1* was knockdown. Furthermore, 45 miRNAs and 13 were reduced significantly only in *HIF1A* or *EPAS1* silencing, respectively (Fig. 4 A, left panel). This pattern was also sustained when we focused on miRNA genes containing HRE motifs in their genomic locations (Fig. 4 A, right panel). This analysis also confirmed CoCl_2_ preference for HIF-1α, since when *HIF1A* was silenced 77 miRNAs were reduced, whereas only 10 (out of 21) were downregulated in the *EPAS1* knockdown (Fig. 4B, left panel). The analysis of HRE motifs also showed that during CoCl_2_ treatments, HIF-1 was the main regulator of miRNA expression (Fig. 4B, right panel). It is critical to note that in DMOG-treated cells, HIFs knockdowns reduced the expression of at least 45 miRNAs. However, only two miRNAs were downregulated when *HIF1A* or *EPAS1* was silenced. (Fig. 4 C, left panel). Furthermore, despite DMOG-related accumulation of HIF-2α, the majority of affected miRNAs contained HIF-1 specific HREs, while only 3 had the respective HIF-2 consensus sequences (Fig. 4 C, right panel). As shown in Fig. 4DE, we also compared knockdown-related miRNA changes between hypoxia and hypoxia mimetics. Although *HIF1A* silencing led to model-specific changes in miRNA expression, 23 miRNAs were commonly affected by both hypoxia and CoCl₂ treatment, while 10 miRNAs were shared between hypoxia and DMOG. Notably, two miRNAs were consistently reduced across all three conditions (Fig. 4D, left panel). This pattern was reflected in changes of HRE-containing miRNA genes, where 9 were common between hypoxia and CoCl_2_, 4 miRNAs were common between hypoxia and DMOG, and 2 miRNAs that were reduced in all 3 conditions (Fig. 4D, right panel). Notably, the *EPAS1* silencing in the hypoxia mimetics was very different compared to the data from hypoxia given that only 6 miRNAs were common between hypoxia and CoCl_2_, and 5 miRNAs were common between hypoxia and DMOG, and this included 1 miRNA that was reduced in all 3 conditions (Fig. 4E, left panel). Furthermore, the HIF-2 consensus motifs were detected for only 2 miRNAs that were common between hypoxia and CoCl_2_ (Fig. 4E, right panel). Taken together, the hypoxia mimetics, especially DMOG, were ineffective in mimicking the hypoxia-specific impact on miRNA expression.

**Fig. 5:**
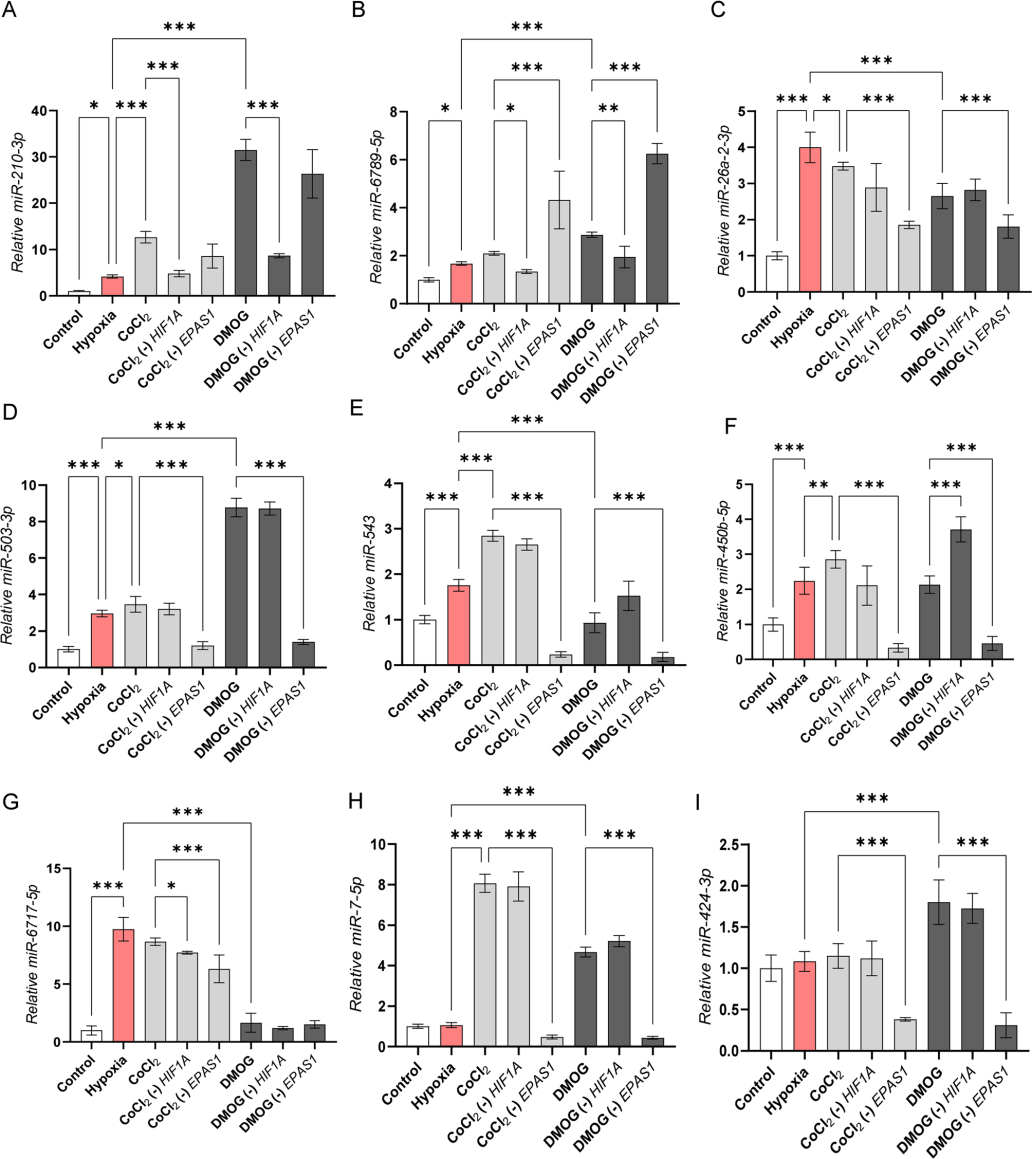
Hypoxia mimetics treatments result in heightened or diminished expression of HIF-1- and HIF-2- related miRNAs compared to hypoxia. The qPCR validation of hypoxia and HIF-regulated miRNA levels that in HUVECs: **A** hsa-miR-210-3p; **B** hsa-miR-6789-5p; **C** hsa-miR-26a-2-3p; **D** hsa-miR-503-3p; **E** hsa-miR-543; **F** hsa-miR-450b-5p; **G** hsa-miR-6717-5p; **H** hsa-miR-7-5p; **I** hsa-miR-424-3p. The validation was performed upon both *HIF-1α* (*-HIF1A*) and *HIF-2α (-EPAS1)* silencing in HUVECs treated with CoCl_2_ or DMOG for 16 h. The cells cultured in normoxia and transfected with control siRNA were used as the normoxic control, whereas cells transfected with control siRNA and exposed to hypoxia for 8 h were included as an additional control. The effects of *HIF1A* and *EPAS1* silencing on these miRNA levels in HUVECs were evaluated previously in [22].miRNA levels were normalized to *RNU44* and expressed as a fold change over control in normoxia. The data represents mean of 3 biological replicates (3 technical replicates each) ± SEM of (*P < 0.05, **P < 0.01 and ***P < 0.001 were considered significant). A summary of the validated miRNA changes upon hypoxia and mimetics exposure, along with their experimentally confirmed targets, is provided in Supplemental Table 2

The global NGS-based selection of expression changes strongly relies on the fold change and the significance thresholds [32], which may overlook some miRNA changes that do not meet these cut-off requirements. To address this issue, we performed an independent qPCR-based verification to determine the impact of the hypoxia mimetics on miRNA expression, focusing on miRNAs that we previously reported to be HIF-dependent, especially those induced by HIF-2 [22]. As shown in Fig. 5A, the expression of miR-210-3p, which is induced during hypoxia by both HIF-1 and HIF-2, was significantly increased in both CoCl_2_ and DMOG treated cells when compared to both control and HUVECs exposed to hypoxia for 8 h. The levels of miR-210-3p were dramatically reduced in cells with the *HIF1A* knockdown. Notably, silencing *EPAS1* had no significant impact on miR-210-3p levels during mimetic treatments (Fig. 5 A). HIF-1-dependent miR-6789-5p was induced by both hypoxia and hypoxia mimetics and reduced upon *HIF1A* silencing, whereas *EPAS1* knockdown resulted in a significant upregulation of this miRNA expression during both CoCl_2_ and DMOG treatments (Fig. 5B). In the case of miR-26a-2-3p, which is proposed to be induced during hypoxia in a HIF-2 dependent manner [22], both mimetics induced expression of this miRNA that was reduced upon *EPAS1* silencing (Fig. 5 C). Hypoxia mimetics also allowed successful confirmation of *EPAS1-*mediated induction of miR-503-3p, although the DMOG treatments resulted in the dramatically higher accumulation of this miRNA than was observed during hypoxia (Fig. 5D). Furthermore, using mimetics we were also able to successfully confirm HIF-2 impact on miR-543 (Fig. 5E) and miR-450b-5p (Fig. 5 F). Nevertheless, the induction of miR-543 by CoCl_2_ and DMOG was significantly higher and lower, respectively, when compared to hypoxia (Fig. 5E), while miR-450b-5p was elevated by *HIF1A* silencing in the DMOG-treated cells (Fig. 5 F). We also verified the mimetics’ impact on miR-6717-5p that was significantly induced under hypoxia, whereas data from CoCl_2_ treatment supported this miRNA induction and suggested that it may be related to both HIF-1 and HIF-2 transcriptional activity, while DMOG treatment did not affect this miRNA’s expression level (Fig. 5G).

We also tested the responses to mimetics for miRNA that were HIF-2-dependent and enriched in RNA-induced silencing complexes (RISCs) of hypoxic HUVECs, which included miR-7-5p and miR-424 that were not induced globally [22] (Fig. 5HI). Silencing *EPAS1* in both DMOG and CoCl_2_ treated cells dramatically reduced expression of these miRNAs, and this is in good agreement with our previous studies [22]. However, miR-7-5p was dramatically induced upon both mimetics’ treatment, whereas miR-424-3p levels were only elevated by DMOG (Fig. 5HI).

**Fig. 6:**
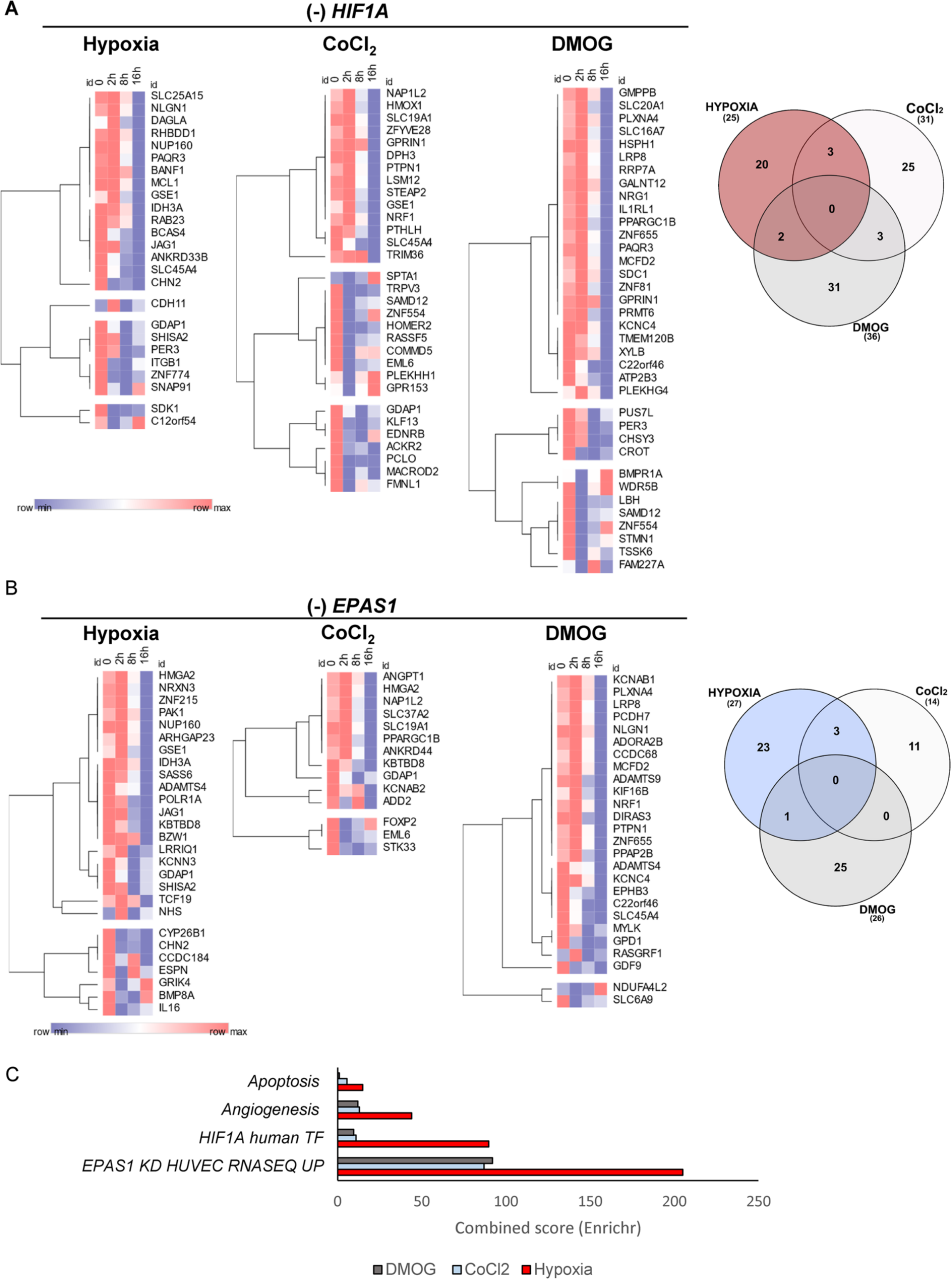
The predicted differences in miRNA-related cellular responses between hypoxia and hypoxia mimetics based on their mRNA target expression during the hypoxia time course. The miRNA-mRNA target interactions were analyzed with the use of miRDIP web server. Only miRNA targets that have been significantly upregulated (*P* value < 0.05) when HIF1A or EPAS1 were silenced in the respective conditions (hypoxia, CoCl_2_ or DMOG) were considered (Supplemental Data Set 3). The changes in these mRNA expression levels during hypoxia based on gene expression microarrays [20] are presented as heatmaps generated and hierarchically clustered with the Morpheus web server (Morpheus, https://software.broadinstitute.org/morpheus). **A** The expression changes of miRNA targets affected by HIF1A silencing in hypoxia upon CoCl_2_ and DMOG treatments are shown. The Venn diagram represents the distribution of mRNA targets between these 3 models. **B** The expression changes of miRNA targets affected by EPAS1 silencing in hypoxia upon CoCl_2_ and DMOG treatments are shown. The Venn diagram represents distribution of mRNA targets between these 3 models. **C**. The cellular signaling assignments that are associated with the identified miRNA targets using the Enrichr database are shown. No enrichment analysis was performed. Instead, a functional Gene Ontology (GO) assignment was conducted, focusing on HIF-related functional and transcriptional effects predicted by four sub-databases: ARCHS4 TFs Coexpression, TF Perturbations Followed by Expression, Panther 2016, and MSigDB Hallmark 2020. As shown in Supplemental dataset 4, the top five significant assignments from each database were selected using the hypoxia dataset as the reference. These same assignments were then identified for the CoCl₂ and DMOG datasets, regardless of their rank or statistical significance. The “combined score” (x-axis) is calculated by taking the logarithm of the *p*-value from the Fisher exact test and multiplying it by the z-score, and this reflects the deviation from the expected rank [34]

To gain insight into the functional differences of the hypoxia mimetics on HIF-related miRNAs, we searched for these miRNA targets within data obtained from the parallel analysis of global mRNAs expression changes upon *HIF1A* and *EPAS1* silencing in the same samples used for miRNA analysis. For target predictions, we used the miRDIP web server at the strictest setting (P value below 0.01 and confirmed by at least four independent prediction algorithms) [33]. Since our focus was on detecting targets of HIF-related miRNAs, we narrowed our analysis to transcripts that were significantly upregulated in the absence of HIFs (Supplemental Data Set 3), as this upregulation could potentially result from increased mRNA stability due to the depletion of specific miRNAs. Finally, to determine the results on the functional effects, we considered only targets that were significantly reduced during hypoxia (by at least log2FC = − 1, *P* value below 0.05) as measured in HUVEC cells [20]. This approach resulted in selection of genes whose expression may be reduced under hypoxia due to the HIF-1- or HIF-2-related miRNA inductions. Notably, targets of hypoxia-mimetic-regulated miRNAs showed minimal overlap with mRNAs altered by hypoxia-induced HIF-1/HIF-2 activity as shown in Fig. 6 (Fig. 6 A represents the effects of *HIF1A* silencing while Fig. 6B shows the consequences of *EPAS1* silencing). These genes were further mapped to signaling pathways using our established method (Fig. 6 C) [20].

The results indicated that CoCl_2_ and DMOG treatment-related miRNA changes are not comparable to the hypoxia HIF-induced miRNA’s impact on angiogenesis, apoptosis, and HIF-1 signaling, although they are with good agreement with the independent report of *EPAS1* silencing in HUVECs [35].

## Discussion

In this study, we provide an in-depth exploration of the utility and limitations of CoCl_2_ and DMOG as hypoxia mimetics for studying HIF-regulated miRNA expression and the related cellular pathways. Our study integrates data from NGS-based global analyses, qPCR validation, and silencing experiments. This allows us to uncover the distinct profiles of HIF-1 and HIF-2 activity induced by these mimetics. Despite their selective efficacy, the findings emphasize significant discrepancies in their ability to replicate hypoxic conditions.

Our central focus was to test the ability of CoCl_2_ and DMOG to mimic the hypoxic transition from HIF-1α to HIF-2α in cultured human endothelial cells [20] as well as in the transition that is observed in some tumors [5]. In contrast to the hypoxia transition, CoCl_2_ and DMOG treatments result in a differential stabilization of HIF-1α and HIF-2α, with CoCl_2_ predominantly stabilizing HIF-1α, and DMOG inducing a prolonged stabilization of HIF-2α. Furthermore, neither mimetic accurately replicates the sequential stabilization observed under physiological hypoxia, and this limits their application as models for dynamic HIF responses. While CoCl_2_ and DMOG show potential in mimicking certain isoform-specific effects, their inability to reflect the simultaneous HIF-α activity seen during hypoxia remains a notable limitation.

The global changes in miRNA expression profiles induced by mimetics further underscore their limitations. while there is some overlap with hypoxia-induced profiles, mimetics-driven changes are more similar to each other than to physiological hypoxia. However, based on our previous findings and distribution of HRE elements in miRNA promotor regions [20, 23], we expected that silencing either of HIF-1α or HIF-2α in hypoxia exposed cells should have a limited impact on related miRNA profiles given that the majority of miRNAs are potentially targets of both these HIF isoforms. Despite the fact that DMOG is considered to be among the most reliable hypoxia mimetic [11, 36], it was much less effective in mimicking the HIF-mediated reprograming of miRNA expression profiles than CoCl_2_.

Furthermore, although CoCl_2_ treatments showed preference for HIF-1 -regulated miRNAs, DMOG treated cells exhibited changes in both HIF-1- and HIF-2-related miRNAs. The impact of DMOG on HIF-1-related miRNAs is likely a result of the compensatory effect of significantly higher HIF-2α levels compared to those in hypoxia-exposed cells. These higher levels, along with the highly similar HIF-1 and HIF-2 consensus motives, suggest that HIF-2 can potentially take over some of the HIF-1 functions. Furthermore, our data suggest that gene expression profiles observed at the 16-hour timepoint following DMOG treatment may reflect the earlier HIF stabilization dynamics, particularly between 6 and 10 h, when both HIF-1α and HIF-2α are present at more balanced levels. This temporal disconnect likely contributes to the predominance of HIF-1-dependent transcriptional features observed at 16 h, despite the apparent HIF-2α stabilization at that time. Notably, transfection procedure, especially the *EPAS1* siRNA treatment, appear to induce compensatory HIF-1α accumulation in DMOG-treated cells, a phenomenon not observed in the CoCl₂-treated or the non-transfected cells. This suggests that transfection itself may influence HIF-α stabilization patterns, adding complexity to the interpretation of gene expression data. While DMOG treatment shares some similarities with hypoxia in terms of the HIF stabilization kinetics, the miRNA profiles do not fully recapitulate those seen under hypoxic conditions [22]. These findings underscore the importance of considering both temporal dynamics and experimental context when interpreting HIF isoform-specific gene regulation.

Furthermore, our HRE analysis of miRNA genes were limited to open chromatin regions in HUVECs that were cultured in normoxic conditions, whereas the hypoxia exposure modifies the chromatin organization [37, 38]. The distinct chromatin remodeling has also been observed with the use of hypoxia mimetics since these compounds have been shown to differentially modulate histone post-translational modifications [17]. Nevertheless, our data suggests a skewed representation of the HIF-mediated miRNA reprogramming, emphasizing the selective but incomplete nature of mimetics-induced hypoxic remodeling.

That being said, in our previous study using qPCR to examine HIF-1 and HIF-2 transcriptional targets in HUVECs, we found that DMOG and CoCl_2_ were effective and consistent tools for confirming the predefined HIF-dependent gene set [23]. Therefore we used a similar approach against hypoxia verified HIF-regulated miRNAs [22]. This qPCR-based validation added a layer of specificity to NGS observations. For instance, the induction of miR-210-3p, a key hypoxia-responsive miRNA by both CoCl_2_ and DMOG, has much higher levels than during hypoxia. The dependency of miR-210-3p on HIF-1α, as opposed to HIF-2α, during mimetic treatments highlights the isoform-specific regulation. Similarly, the identification of HIF-2-specific miRNAs, such as miR-503-3p and miR-543, underscores the selective efficacy of DMOG. These approaches confirmed the presence of isoform specific-specific regulation in other miRNAs, including miR-6789-5p (HIF-1-dependent) and miR-503-3p (HIF-2-dependent). However, the heightened or diminished expression of these miRNAs compared to hypoxia raises concerns about the physiological relevance of these mimetics-induced profiles. Overall, these results establish that the mimetics are as a valuable tool to expand research on HIF-related miRNAs. However, mimetics cannot replicate the cooperative regulation observed under hypoxia and limits their application in studying the full spectrum of HIF-miRNA interactions.

The functional consequences of HIF-regulated miRNA expression are profound given their influence on pathways such as angiogenesis, apoptosis, and metabolic reprogramming [10, 20]. Thus our investigation also delves into predicting the functional consequences of HIF-mediated postranscriptional regulation of gene expression by linking miRNA expression changes to their mRNA targets. Notably, these analyses of HIF-related miRNA targets reveal that mimetics-induced changes do not fully overlap with hypoxia-induced effects. This discrepancy is particularly evident in pathways related to angiogenesis and apoptosis, where mimetics fail to replicate the nuanced regulation observed under physiological hypoxia.

Notably, the small-molecule prolyl hydroxylase inhibitors recently developed for clinical use in renal anemia—such as Roxadustat, Molidustat, and Daprodustat—not only offer higher specificity for PHD enzymes, but also more accurately replicate the kinetics of HIF-1α and HIF-2α accumulation [39]. As such, they may provide more reliable insights into HIF-mediated transcriptomic and post-transcriptomic reprogramming. However, dedicated studies are needed to fully evaluate their utility as experimental hypoxia mimetics.

Importantly, cell culture-based hypoxia models, including the one presented here, are inherently limited by the challenge of defining appropriate normoxic controls [40]. Most in vitro studies rely on near-atmospheric oxygen levels (16–18% O₂), which are substantially higher than physiological oxygen concentrations (physioxia). As shown by Alva et al., was significantly influenced by the baseline oxygen conditions [41]. Notably, these cancer cells cultured under standard conditions (18% O₂) exhibited enhanced induction of HIF target genes upon hypoxia exposure compared to those preadapted to 5% O₂ [41]. This suggests that conventional culture conditions may exaggerate hypoxic responses. However, replicating physioxia in cell culture remains highly challenging due to multiple factors affecting pericellular O₂ concentration, including media height and type, cell-specific oxygen demands, seeding density, and the proliferation rate [42, 43]. Therefore, developing a model that accurately reflects physioxic conditions for HUVECs could provide deeper insights into HIF-mediated transcriptional reprogramming and endothelial cell physiology.

Taken together, while CoCl_2_ and DMOG offer some valuable insights into the isoform-specific activity of HIF-1 and HIF-2, their inability to fully replicate the complexity of hypoxia-induced HIF-miRNA interactions necessitates caution when interpreting results.It is only by integrating and verifing mimetics-derived data with findings from low oxygen-induced hypoxia models that the intricacies of hypoxic adaptation at the post-transcriptional level can be unraveled.

## Materials and methods

### Cell culture

 Primary human umbilical vein endothelial cells (HUVECs) that were pooled from ten individual donors were purchased from Cellworks (Caltag Medsystems Ltd, UK) and cultured in EGM-2 Bulletkit Medium (Lonza). All experiments were conducted between passage 2 and 6 at a confluence of 70–80%.

### Induction of hypoxia

 Hypoxia was induced in a CO_2_/O_2_ incubator/chamber specific for hypoxia research (InvivO2 Baker Ruskinn). Briefly, cells were cultured in 35–60 mm dishes for RNA isolation and protein isolation, respectively, at 0.9% O_2_ for the time periods specified (PO_2_ was 6.84 mm Hg). Control cells were maintained in normoxia in a CO_2_ incubator (Thermo Fisher Scientific). The hypoxia mimetics, Dimethyloxalylglycine; N-(Methoxyoxoacetyl)-glycine (DMOG, Sigma, D3695) and CoCl_2_ (Sigma, 60818), were used at final concentrations of 1 mM and 200 µM for the time periods specified.

### siRNA transfections

 siRNA for *HIF1A*, (Ambion assay id s6539) and *EPAS1* (Ambion assay id s4698), were purchased from Ambion. HUVECs were transfected using the Lipofectamine RNAiMax (Invitrogen, 13778030) according to manufacturer’s protocol. The siRNAs were used at a final concentration of 20 nM. The transfected cells were cultured for 2 days prior to further analysis. Ambion siRNA Negative Control 1 (Ambion assay id MC22484) was used as a control. After 24 h, the transfected cells were put into a hypoxia chamber or treated with CoCl_2_ or DMOG, whereas the control cells remained in an incubator in normoxic conditions.

### Isolation of RNA

 The cells were lysed directly on culture vessels with QIAzol lysis reagent (Qiagen). Total RNA (containing both mRNA and miRNA) was isolated using miRNeasy kit (Qiagen). RNA concentrations were calculated based on the absorbance at 260 nm. RNA samples were stored at −70 °C until use.

### Measurement of miRNA and mRNA using quantitative real time PCR (qRT-PCR)

TaqMan One-Step RT-PCR Master Mix Reagents (Applied Biosystems) was used as described previously [44–48] using the manufacturer’s protocol (retrotranscription: 15 min, 48 °C). The relative expressions were calculated using the 2^−ΔΔCt^ method [49] with the Ribosomal Protein Lateral Stalk Subunit P0 (*RPLP0*) and *18 S rRNA* genes as reference genes for the mRNA, and *RNU48* and *RNU44* were used as references for the miRNA. TaqMan probe ids used are given in the Supplemental Table 1.

### Western Blots

 Western Blot analysis was performed as previously described [48]. Briefly, cells were lysed in SDS lysis buffer (4% SDS, 20% glycerol, 125 mM Tris–HCl pH = 6.8) supplemented with protease inhibitors (cOmplete ™ Mini, Roche). The insoluble material was removed by centrifugation at 15,000 g for 15 min. Protein concentrations were determined by Bio-Rad™ DC-Protein Assay using bovine serum albumin (BSA) as standard. Following the normalization of the protein concentrations, the lysates were mixed with an equal volume of 6X Laemmli sample buffer (12% SDS, 60% glycerol, 0.06% bromophenol blue, 375 mM Tris-HCl pH = 6.8) and incubated for 5 min at 95 °C prior to separation by SDS-PAGE on a 4–15% Criterion TGX Stain-Free Gel (Bio-Rad, Hercules, CA, USA). Following SDS-PAGE, the proteins were transferred to polyvinylidene fluoride membranes (Bio-Rad) using the wet electroblotting method (300 mA, 4 °C, 90 min for one gel and 180 min for two gels). The membranes were blocked with BSA dissolved in TBS/Tween-20 (5% BSA, 0.1% Tween-20 overnight at 4 °C), followed by immunoblotting with the primary antibodies (2 h) that included mouse anti–HIF-1α (1:2000, ab16066; Abcam), rabbit-anti-HIF-1a D1S7W (1:1000, #36169; Cell Signaling), rabbit anti–HIF-2α (1:1000, ab199; Abcam), rabbit anti-HIF-2a D6T8V (1:1000, #59973: Cell Signaling), rabbit anti-actin (1:1000, #4967; Cell Signaling). After the washing steps, the membranes were incubated with goat anti-rabbit IgG (heavy and light chains) or with goat anti-mouse IgG (heavy and light chains) horseradish peroxidase-conjugated secondary antibodies (Bio-Rad) for 1 h at room temperature and detected using SuperSignal West Pico ECL (Thermo Scientific). Densitometry was performed using the Image Lab software v.4.1 (Bio-Rad).

### Next generation RNA sequencing

HUVECs were used for RNA isolation and analyses. Following total RNA isolation, samples were validated with quantitative real-time PCR for activation of the hypoxic response prior to further analysis. Following rRNA depletion, the remaining RNA fraction was used for library construction and subjected to 75-bp paired-end sequencing on an Illumina NextSeq 500 instrument (San Diego, CA, USA). Sequencing reads were aligned to the Gencode human reference genome assembly (GRCh38 p13 Release 32) using STAR version 2.7.3a [50]. Transcript assembly and estimation of the relative abundance and tests for differential expression were carried out with Cufflinks and Cuffdiff [51]. Transcripts that showed a statistically significant change (*P* value < 0.05) and a log2 fold change of at least ± 1 were selected. The resulting data were validated with quantitative real-time PCR.

Small RNA sequencing libraries were prepared using QIAseq miRNA library kit (Qiagen) following the manufacturer’s instructions and followed by sequencing on an Illumina NextSeq 500 instrument. Using Qiagen’s Gene Globe Software, sequencing reads were aligned to the human reference genome assembly (hg19) followed by transcript assembly and estimation of the relative abundances. The analysis of the differential expression of small RNAs between control and experimental samples were performed with geNorm [52] in the Gene Globe Software. Low read miRNA changes (below 10) were ignored.

The heatmap generation and hierarchical clustering were performed with the Morpheus Webserver (https://software.broadinstitute.org/morpheus*).* The Enrichr Webserver (https://amp.pharm.mssm.edu/Enrichr/*)* [34] was applied to assign the NGS results into the “Gene Ontology " categories with the selection based on a *Q* value < 0.05. The cellular signaling assignments that are associated with the identified miRNA targets using the Enrichr database are shown. No enrichment analysis was performed. Instead, a functional Gene Ontology (GO) assignment was conducted, focusing on HIF-related functional and transcriptional effects predicted by four sub-databases: ARCHS4 TFs Coexpression, TF Perturbations Followed by Expression, Panther 2016, and MSigDB Hallmark 2020. As shown in Supplemental dataset 4, the top five significant assignments from each database were selected using the hypoxia dataset as the reference. These same assignments were then identified for the CoCl₂ and DMOG datasets, regardless of their rank or statistical significance. The “combined score” (x-axis) is calculated by taking the logarithm of the p-value from the Fisher exact test and multiplying it by the z-score, and this reflects the deviation from the expected rank [34]. Finally, the miRNA-mRNA target interactions were analyzed with the use of miRTarBase [53]. The microRNA Data Integration Portal (mirDIP, https://ophid.utoronto.ca/mirDIP/) was used with the bidirectional search mode at confidence class “high” that represents the top 5% of potential interactions (*P* value *< 0.05*) [33].

### Hypoxia Response Element (HRE) analysis

 The promoters of the miRNAs were analyzed for HIF-1 or HIF-2 binding sites. In each promoter sequence that was defined as a 20 kb window around the transcription start site (TSS), only the open chromatin regions were examined that were established in the HUVEC cell line by the ENCODE [54] project. Both DNase I-seq HUVEC datasets found in Ensemb l were merged [55] (v.79). The focus was on two distinct HRE motifs annotated to HIF-1 (M00139, alias *HIF1A)* and HIF-2 (M00074, alias *EPAS1)* from the HOCOMOCO v.9 database [56]. We note that from the version 10 onwards, the creators of HOCOMOCO have decided to choose a single generic model for each TF [56], resulting in assignment of nearly identical motifs to HIF1A and *EPAS1*, however, based on previous research reports [26–29], we continue to use the two distinct HRE motifs annotated to *HIF1A* and *EPAS1*. The Nencki Genomics Database [57] (v. 79_1) was used to obtain genomic coordinates of these motif instances. For each miRNA, we calculated the number of instances found in the open chromatin regions for both HIF-1 and HIF-2.

### Statistical analysis

 Results were expressed as a mean ± standard deviation (SD). Statistical significance was determined using the Student’s t-test and ANOVA and a *P* value < 0.05 was considered significant. The correlations were accessed via the Spearman rank–order correlation method.

## Supplementary Information

Supplementary Material 1: Supplemental Fig. 1. The representative changes in HIF-1α and HIF-2α protein levels were evaluated after exposure to hypoxia or treatments 200 µM CoCl_2_ and 1mM DMOG for the time periods specified by Western Blot. The uncropped Western blots are presented that include non-related samples.

Supplementary Material 2.

Supplementary Material 3.

Supplementary Material 4.

Supplementary Material 5.

Supplementary Material 6.

Supplementary Material 7.

## Data Availability

Deep sequencing data were deposited in [Gene Expression Omnibus](http://www.ncbi.nlm.nih.gov/geo) (GEO) at accession numbers: GSE116909, GSE190240 and GSE190242. The accompanying data are included in supplemental materials or available on request.

## Abbreviations

DMOG: Dimethyloxalylglycine
*EPAS1*: Hypoxia inducible factor 2 alpha
FIH: Factor inhibiting HIF
HIF: Hypoxia inducible factor
*HIF1A*: Hypoxia–inducible factor 1 alpha
HIF-1: Hypoxia–inducible factor 1
HIF-2: Hypoxia–inducible factor 2
HUVECs: Human umbilical vein endothelial cells
HRE: Hypoxia response element
miRNA: MicroRNA
NGS: Next generation sequencing
PHD: Prolyl hydroxylase
qPCR: Quantitative polymerase chain reaction
RISC: RNA–induced silencing complex
*RPLP0*: Ribosomal Protein Lateral Stalk Subunit P0
SD: Standard deviation
SEM: Standard error of the mean
siRNA: Small interfering RNA TF–Transcription factor
TSS: Transcription start site
