# Supplementary material for: Challenges in mimicking hypoxia: insights into HIF-regulated MiRNA expression induced by DMOG and CoCl2
Source: Cell Commun Signal. 2025 Oct 22;23:454. doi: 10.1186/s12964-025-02459-7 (PMC12542114; doi:10.1186/s12964-025-02459-7)
Supplement: Supplementary file 6 — Supplementary Material 6. [file 12964_2025_2459_MOESM6_ESM.docx]

**Supplemental Table 1.** TagMan Probes IDs used in the measurement of miRNA and mRNA quantitative Real Time PCR.

|  | **Assay Name** | **Assay ID** |
| --- | --- | --- |
| **Gene Expression Assay** | *18S* | Hs99999901_s1 |
|  | *TBP* | Hs00427620_m1 |
|  | *RPLP0* | Hs00420895_gH |
|  | *HIF1A* | Hs00153153_m1 |
|  | *EPAS1* | Hs01026149_m1 |

| **MicroRNA Assay** | RNU44 | 001094 |
| --- | --- | --- |
|  | RNU48 | 001006 |
|  | hsa-miR-10a-3p | 002288 |
|  | hsa-miR-139-5p  (bta-miR-139) | 005364_mat |
|  | hsa-miR-210-3p | 000512 |
|  | hsa-miR-26a-2-3p | 002115 |
|  | hsa-miR-342-5p | 002147 |
|  | hsa-miR-374a-3p | 002125 |
|  | hsa-miR-424-3p | 002309 |
|  | hsa-miR-450b-5p | 002207 |
|  | hsa-miR-4745-5p | 464126_mat |
|  | hsa-miR-495-3p | 001663 |
|  | hsa-miR-503-3p | 476380_mat |
|  | has-miR-503-5p | 001048 |
|  | hsa-miR-520d-3p | 002743 |
|  | hsa-miR-543 | 002376 |
|  | hsa-miR-6789-5p | 466828_mat |
|  | hsa-miR-7-5p | 005723_mat |
|  | hsa-miR-98-3p | 472125_mat |
